# P10 Ceftazidime/avibactam for OXA-48 urinary tract infections—a 12 month remissive study

**DOI:** 10.1093/jacamr/dlad077.014

**Published:** 2023-08-02

**Authors:** Afonso Paes Fernandes, Beatriz Silva, Ana Coutinho

**Affiliations:** Hospital de Cascais, Portugal; Hospital de Cascais, Portugal; Hospital de Cascais, Portugal

## Abstract

**Background:**

Ceftazidime/avibactam is a novel antibiotic for MDR bacterial infections. Despite its high cost, from 1 January 2022 to 31 December 2022, 23 patients out of 230 hospital beds at the Hospital of Cascais received it. Seven of these patients had OXA-48-producing urinary tract infections (UTIs). One was a post-stroke ICU patient with a urinary catheter; all others were in the urology unit, with no invasive devices.

**Objectives:**

To assess appropriateness, efficacy and renal toxicity of ceftazidime 2000 mg + avibactam 500 mg in the seven UTI patients with isolation of OXA-48-producing bacteria in urine samples.

**Methods:**

From patient history, lab analysis and drug chart we assessed appropriateness—by meropenem (alternative therapy if MIC <16.000 mg/L); efficacy—from overall outcomes; renal toxicity—by evaluating serum creatinine progression (the rationale being SPC describes increases in serum creatinine as a frequent adverse reaction). We also describe dose and days of therapy.

**Results:**

All had antibiotic adequacy, two meropenem MICs <16.000 mg/L were found but carbapenem therapy was not recommended due to drug interaction or allergy. Median days of therapy was 8.7 days. Four outcomes were successful, the ICU patient had a death not related to infection, one urology unit patient died and in one patient treatment was not successful—OXA-48 was still detected in urine samples after 7 days of therapy. No renal toxicity from ceftazidime/avibactam was noted.

**Conclusions:**

Ceftazidime/avibactam prescription in UTIs has been adequate, safe and efficient in some patients. Literature describes that efficacy of ceftazidime/avibactam varies with kidney status. Kinetics is known to be linear, so therapeutic drug monitoring will be the way forward. Further study will be necessary to address definitive safety and efficiency.

